# Supplementary material for: Mechanically Tough and Highly Stretchable Hydrogels Based on Polyurethane for Sensitive Strain Sensor
Source: Polymers (Basel). 2023 Sep 27;15(19):3902. doi: 10.3390/polym15193902 (PMC10575362; doi:10.3390/polym15193902)
Supplement: Supplementary file 1 [file polymers-15-03902-s001.zip › polymers-2595299-supplementary.pdf]

Supporting Information

Mechanically Tough and Highly Stretchable Hydrogels Based on Polyurethane for Sensitive Strain Sensor

Jianyang Shi [1,2], Shuang Wang [2], Haibo Wang [1,2] and Jun Gu [1,2,*]

[1] Department of Cardiovascular Surgery, West China Hospital, Sichuan University, Chengdu 610065, China; sjy812001920@163.com (J.S.); whb6985@scu.edu.cn (H.W.)

[2] College of Biomass Science and Engineering, Sichuan University, Chengdu 610065, China;

shuangshine7@scu.edu.cn

[*] Correspondence: gujun@wchscu.cn; Tel.: +86-28-85401296

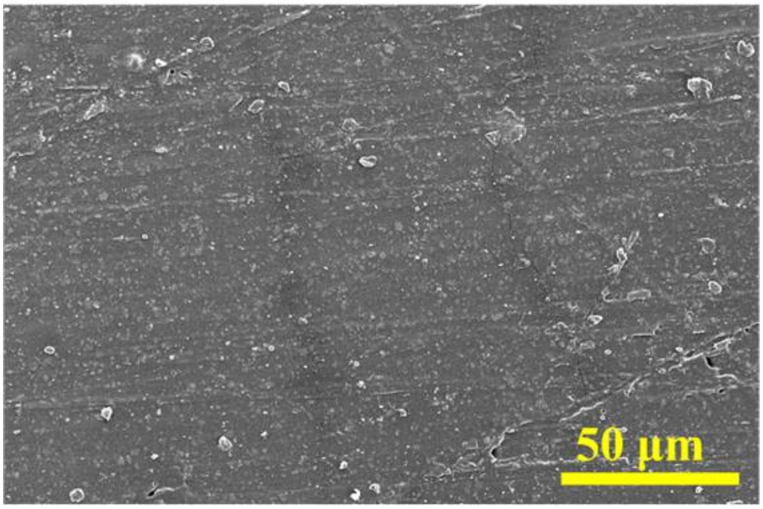

Figure S1. Cross-sectional SEM images of lyophilized PCA.

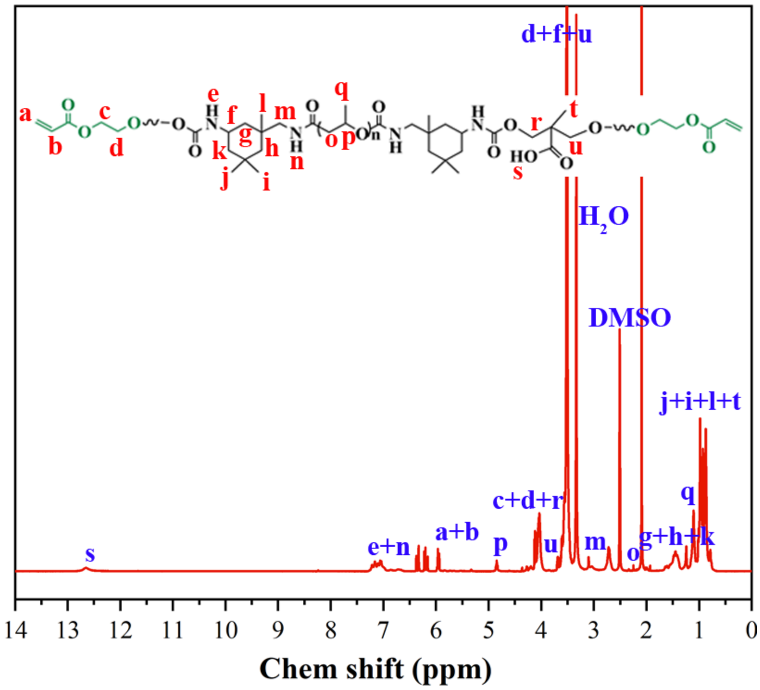

Figure S2. The 1H NMR spectrum of PCA (DMSO-d_6).
